# Prevention of mother to child transmission of HIV in Tanzania: assessing gender mainstreaming on paper and in practice

**DOI:** 10.1093/heapol/czx080

**Published:** 2017-07-14

**Authors:** Tumaini Nyamhanga, Gasto Frumence, Daudi Simba

**Affiliations:** 1 Department of Development Studies, Muhimbili University of Health and Allied Sciences, Dar es Salaam, Tanzania and; 2 Department of Community Health, Muhimbili University of Health and Allied Sciences, Dar es Salaam, Tanzania

**Keywords:** PMTCT, HIV, gender mainstreaming

## Abstract

Although gender mainstreaming has been long recognized as a strategy for addressing gender inequalities and associated negative health outcomes; its implementation has remained a challenge, even in the area of prevention of mother to child transmission of HIV (PMTCT). Despite recognition of gender in Tanzania’s political arena and prioritization of PMTCT by the health sector, there is very little information on how well gender has been mainstreamed into National PMTCT guidelines and organizational practices at service delivery level. Using a case study methodology, we combined document review with key informant interviews to assess gender mainstreaming in PMTCT on paper and in practice in Tanzania. We reviewed PMTCT policy/strategy documents using the WHO’s Gender Responsive Assessment Scale (GRAS). The scale differentiates between level 1 (gender unequal), 2 (gender blind), 3 (gender sensitive), 4 (gender specific), and 5 (gender transformative). Key informant interviews were also conducted with 26 leaders purposively sampled from three government health facilities in Mwanza city to understand their practices. The gender responsiveness of PMTCT policy/strategy documents varies, with some being at GRAS level 3 (gender sensitive) and others at GRAS level 4 (gender specific). Those which are gender sensitive indicate gender awareness, but *no* remedial action is developed; while those which are gender specific go beyond indicating how gender may hinder PMTCT to highlighting remedial measures, such as the promotion of couple counselling and testing for HIV. In addition, interviews on organizational processes and practices suggested that there has been little attention to the holistic integration of gender in the delivery of PMTCT services. The study has revealed limited integration of gender concerns in PMTCT policy documents. Similarly, health facility leader responses indicate perspectives and practices that pay little attention to the holistic integration of gender in the delivery PMTCT services.

Key Messages

- Despite being important for reproductive and child health, gender mainstreaming has not received adequate attention in PMTCT policy documents and organizational practices at service delivery level.
- Most PMTCT policy/strategy documents are at level 3 of gender responsiveness—categorized as gender sensitive**.** That is, the documents’ content indicates awareness of gender concerns, but lack corresponding remedial measures.
- Health facilities’ organizational processes and practices pay little attention to holistic integration of gender in the delivery of PMTCT services.

## Introduction

Globally, 3.2 million children under 15 are infected with HIV, of whom 91% live in sub-Saharan Africa (UNAIDS 2014). This alarming situation is partly explained by the fact that sub-Saharan Africa is home to nearly 90% of pregnant women living with HIV (UNAIDS 2011). In 2011, UNAIDS launched a global plan to enable 21 sub-Saharan African countries with the highest burden of paediatric HIV to eliminate new HIV infections by 2015 (UNAIDS 2011); Tanzania is among these countries. Although substantial progress has been made (48% decline in new HIV infections among children), none of the 21 priority countries achieved the set target. For instance, in 2014 Tanzania had 84 000 pregnant women living with HIV who delivered and recorded 7200 new paediatric HIV infections (IATT 2015).

The risk of maternal to child transmission (MTCT) of HIV is influenced by the extent of women’s vulnerability to HIV. This is more so in sub-Saharan African countries, including Tanzania, where HIV transmission is predominantly heterosexual (Heggenhougen and Lugalla 2005). It follows that gender—which refers to socio-culturally constructed roles, duties, rights, opportunities, accepted behaviours, and status of women and men in relation one another—is a key social determinant of the epidemic among women and children in this region (Shisana et al. 2010; Ramjee and Daniels 2013). In some communities, for example, gender-related cultural and socio-economic factors interact with sexual behaviour and practices to allow for HIV transmission (WHO 2014). Specifically, women’s income poverty, resulting from limited ownership of economic resources and low educational achievement, makes it difficult for them to avoid risky sexual practices. This socio-economic handicap is coupled with cultural attitudes that see men—as husbands and household heads—as having full control of the sexuality of their wives/of women; and that women are expected to provide sexual satisfaction to men and have little power to negotiate safer sex. Women who rebel against this expectation are physically or emotionally abused and consequently succumb to risky non-consensual sex (Dunkle et al. 2004).

Violence experienced by women is based on a number of factors—such as women’s low status, economic dependence on the male partner, alcohol intoxication by the male partner, marital infidelity and socio-culturally constructed gender norms about the ‘proper’ roles and responsibilities of men and women (Jewkes 2002; Kim and Motsei 2002; Stockl et al. 2010; Heise 2012). These norms socialize boys and men to be aggressive, powerful, unemotional, and controlling, there by leading to societal approval of men as dominant partners (Jakupcak et al. 2002). Additionally, these masculine norms lead men to believe that women and children are a man’s possessions and under his control (Jakupcak et al. 2002). Conversely, girls and women are socialized to be passive, nurturing, submissive, and emotional—qualities which inculcate a social acceptance of powerlessness (Scott et al. 2013). These differing patterns of socialization result in an unequal power relationship between men and women (Strebel et al. 2006), which in turn increases women’s vulnerability to HIV.

The influence of gender-related factors on women’s vulnerability to HIV extends to that of her baby. The mother’s financial constraints, the threat of gender based violence on disclosure of HIV status, stigma associated with non-breastfeeding, and limited male involvement, impairs efforts to protect children from acquiring HIV infection during and after delivery. It follows that the risk of maternal to child transmission of HIV is heightened in regions with high levels of gender inequity (Strebel et al. 2006). Thus, for an HIV/AIDS intervention strategy to succeed, gender-related factors fuelling MTCT need to be addressed. These factors cannot be adequately addressed, however, unless gender is mainstreamed into prevention of mother to child HIV transmission policies and programmes.

Gender mainstreaming—which refers to a strategy to advance gender equality by making women’s as well as men’s concerns and experiences an integral dimension of the design, implementation, monitoring and evaluation of policies and programmes—serves to increase effectiveness and coverage of the interventions (UNECOSOC 1997: e1). In the context of PMTCT, gender mainstreaming involves conducting a gender analysis with a view of understanding the cultural and social factors that increase vulnerability to HIV infection and those that limit uptake of antiretroviral therapy and adherence to safe infant feeding. Solutions are then developed to address the identified barriers. Despite its importance, there is little understanding on how well gender has been mainstreamed into national PMTCT guidelines and leadership process and practices at the service delivery level. To fill this gap, this study explored the gender responsiveness of Tanzania’s PMTCT national policy/strategy documents and leadership processes and practices in relation to PMTCT service delivery.

## Methods

### Data collection

A total of five PMTCT policy/strategy documents were reviewed and analyzed (Table 1). These were obtained in hard/soft forms from the Ministry of Health and Social Welfare, and consisted of all PMTCT policy documents that could be obtained from the Ministry.

**Table 1.** Documents reviewed

Policy/strategy documents reviewed	National Scale up Plan for The Prevention of Mother to Child Transmission of HIV and Paediatric HIV Care and Treatment, 2009–2013 National Guidelines for Comprehensive Care Services for Prevention of Mother to Child Transmission of HIV and Keeping Mothers Alive, 2013. Tanzania Elimination of Mother to Child Transmission of HIV Plan, 2012–2015. National Communication Strategy for the Elimination of Mother to Child Transmission of HIV [eMTCT], (2014–2017). National Training Refresher Package. Services for Comprehensive Care and Prevention of Mother-to-Child Transmission. Participant Manual. 2013.

In addition to reviewing the PMTCT policy/strategy documents, 26 key informant interviews were conducted at three government health facilities in Mwanza City. A stratified purposive sampling technique was employed to recruit leaders of the selected health facilities (two hospitals and a health centre), and heads of respective reproductive and child health units involved in PMTCT (Table 2).

**Table 2.** Distribution of respondents

Health facility	Medical officer in-charge	Cadre of the leader						Total
		Head of Nursing services	Head of Antenatal clinic	Labour ward in-charge	Head of Reproductive and child health clinic	Head of Voluntary Counselling and Testing (VCT) units	Head of Family Planning Unit	
Health Facility A	1	1	1	1	1	4	1	10
Health Facility B	1	1	1	1	1	3	1	9
Health Facility C	1	1	1	1	1	1	1	7
Total	3	3	3	3	3	8	3	26

Interview questions were composed around four elements of the comprehensive approach to PMTCT as stated in the national guidelines for prevention of mother to child transmission of HIV and keeping mothers alive (MoHSW 2013). They are: primary prevention of HIV among women of childbearing age and their partners; prevention of unintended pregnancies among women living with HIV; prevention of vertical transmission of HIV from mothers to their infants; and provision of treatment, care and support to women living with HIV and their partners, infants, and families.

### Ethical considerations

Ethical approval was obtained from the authors’ institute. In addition, permission to collect data was sought from the Regional and District level government authorities. Informed consent for participating in the research was sought by the Principal Investigators or research assistants. Each potential respondent was informed of the objectives and significance of the study, as well as the potential benefits and risks of participating in the study. Further, in order to maintain confidentiality, data was gathered without names and unique identifiers were attached to the data and only known to the researchers. Each potential respondent was informed about his/her right to decline participation outright, or to withdraw consent at any stage of the research, without undesirable consequences. The information that was explained to the respondent by the research assistant was summarized on the informed consent form, which the respondents consented to by signing.

### Data management and analysis

The government PMTCT documents were organized according to their respective categories—that is, guidelines, frameworks, and strategic plans—and reviewed for gender responsiveness using content analysis (Elo and Kyngas 2008). The documents were read closely to identify and extract any content on gender, such as text related to the influence of gender relations, division of roles, and/or power differences between men and women on vulnerability to HIV infection. For each document, gender related text was copied onto a matrix with three columns: on the first column gender text taken from the document was pasted; the second column had questions composed around the WHO Gender Responsive Assessment Scale (GRAS); and the third column was where the GRAS level of the document was summarized.

The GRAS assesses gender responsiveness in relation to one of five levels. Documents which are gender unequal (GRAS level 1) have content which perpetuates gender inequality by reinforcing unbalanced norms, roles and relations. Moreover, such documents have statements whose implications privilege men over women (or vice versa), often leading to one sex enjoying more rights or opportunities than the other. Documents which are gender blind (GRAS level 2) have content that ignores gender norms, roles and relations and differences in opportunities and resource allocation for women and men.

Documents which are gender sensitive (GRAS level 3) have content which indicates awareness of the impact of gender norms, roles, and relations, but *no* remedial actions are developed. Documents which are gender specific (GRAS level 4) go beyond indicating how gender may hinder PMTCT to highlighting remedial measures, such as the promotion of couple counselling and testing for HIV. Documents which are gender transformative (GRAS level 5) have content which includes ways to transform harmful gender norms, roles and relations. Additionally, such documents contain strategies to foster progressive changes in power relationships between women and men.

Key informant interviews were thematically analyzed using manual coding. The analysis process took the form of four stages: line-by-line coding of field notes and transcripts; examination and interpretation of codes into descriptive themes; condensation of descriptive themes into analytical themes; and development of an overarching theme.

## Results

This section presents findings on the extent of gender responsiveness of PMTCT policy/strategy documents and leadership processes and practices at the point of PMTCT service delivery.

### Gender responsiveness of PMTCT policy/strategy documents

Content analysis of the five PMTC government documents showed that gender-related factors are mentioned in all of them, and, as a result, there is some consideration of gender within government policy. However, gender was only discussed in a few sections, and in many cases not in the key sections. For example, gender was not integrated into the documents’ goals and objectives or strategies. Moreover, the level of gender responsiveness of those parts that did mention gender ranged between GRAS level 3 (gender sensitive) and GRAS level 4 (gender specific). None of the reviewed policy/strategy documents could be graded as gender transformative. While the policy/strategy documents indicate recognition of gender inequality in decision-making and access to resources as a barrier to accessing PMTCT services by women, no attempt is made to transform harmful gender norms, roles, or relations. For example, the policy/strategy documents do not include actions to transform masculine norms which discourage men from seeking care, taking an HIV test, and/or accompanying their wives/partners to PMTCT clinics. Overall, the documents have a low level of gender mainstreaming, with gender only being incorporated into a few sections, and in many cases not including remedial action. A detailed overview of the above findings is presented below.

#### 1. Policy/strategy PMTCT documents

Table 3 showcases the GRAS level of each of the policy/strategy documents. Four of the five documents were graded at level 3, whereas one of the five documents was graded at level 4.

**Table 3.** GRAS level of policy/strategy documents

Policy/strategy document	GRAS level
National Scale up Plan for The Prevention of Mother to Child Transmission of HIV and Paediatric HIV Care and Treatment, 2009–2013	3
Tanzania Elimination of Mother to Child Transmission of HIV Plan, 2012–2015	3
National Guidelines for Comprehensive Care Services for Prevention of Mother to Child Transmission of HIV and Keeping Mothers Alive, 2013	3
National Communication Strategy for the Elimination of Mother to Child Transmission of HIV [eMTCT], (2014–2017)	3
National Training Refresher Package. Services for Comprehensive Care and Prevention of Mother-to-Child Transmission. Participant Manual. 2013	4

##### Level 3: Gender sensitive policy/strategy documents

Tanzania’s PMTCY policy documents have sections acknowledging the influence of gender on PMTCT, but lack sections on remedial measures. The National Guidelines for Comprehensive Care Services for Prevention of Mother to Child Transmission of HIV and Keeping Mothers Alive, 2013 (MoHSW, 2013a), for example, has a section which reads:

*‘The fear of knowing and eventually disclosing their HIV status deters women from seeking PMTCT services and results in poor adherence to PMTCT interventions, in particular safer infant-feeding decisions, decisions on taking and adhering to ARV medication, condom use and family planning and preference not to deliver at healthcare facilities’(pg.22–23).*

Within the National Scale up Plan for The Prevention of Mother to Child Transmission of HIV and Paediatric HIV Care and Treatment, 2009–2013 gender is mentioned as one of the plan’s guiding principles: ‘*Gender imbalances underlie the pattern of response to illness and health care seeking behavior. They also affect how care and other forms of support are provided’(pg.13).* Despite this, gender is not incorporated into the document’s goals, objectives, or strategies. Likewise, the road map/matrices for the scale up plan (by strategic objectives) do not incorporate gender. Thus, while this can be considered gender sensitive, the fact that gender is only mentioned in terms of recognition in the guiding principles, but not in terms of remedial or transformative action when it comes to strategies or scale up plans means that gender responsiveness is actually quite low.

Similar to the above strategy, the Tanzania Elimination of Mother to Child Transmission of HIV Plan, 2012–2015 includes gender as one of the plan’s guiding principles, and uses identical language. The principle reads: ‘*Gender imbalances underlie the pattern of response to illness and health care seeking behavior. They also affect how care and other forms of support are provided’(pg.23).* However, with regard to interventions, the plan pays very little attention to gender norms, roles, and relations. When statements do consider gender, they are often too general to offer effective guidance. For instance, in order to achieve 50% reduction of HIV incidence among women of reproductive age Group (15–49), the strategy states that there will be ‘*provision of quality early antenatal care services and primary HIV prevention’*, and that:

‘*male involvement will be prioritized through a multipronged approach in both facility and community. At the health facility, couple HIV counseling and testing services will be offered and at the community there will be demand creation through village health committees and door to door HIV testing strategy will be used’* (pg.17).

The above quoted statements are not sufficient. They appear to be neutral, ignoring the limiting effects of gender norms, roles and relations.

The National Guidelines for Comprehensive Care Services for Prevention of Mother to Child Transmission of HIV and Keeping Mothers Alive, 2013 has two sections devoted to gender. Within the introduction, for example, there is a section titled: ‘Gender and HIV’. In this section, the document discusses the impact that gender has on differential vulnerability to HIV between men and women. This section points out that biological and cultural factors contribute to higher rates of HIV infection among women; however, it does not describe the ways in which cultural factors contribute to women’s increased vulnerability to HIV.

For instance, the section reads.

‘…Cultural, traditional, and social factors that increase women’s risk of becoming infected with HIV include: early marriage; concurrent multiple sexual partners; lack of sex education; traditional male attitude about sex; coercion by men who have multiple sexual partners…’ pg.14.

Another section highlighting the influence of gender is in chapter 3, titled ‘stigma, gender and PMTCT programmes’. Again, while this section shows awareness of the impact gender may have on PMTCT in relation to stigma, it lacks linkage to broader gender norms, roles and relations**:**

*‘As a consequence of HIV-related stigma, women may experience violence, loss of shelter and economic support, and exclusion from their family and community. Fear of social stigma; abandonment by family, friends and community; and extreme feelings of isolation and loneliness, as well as the perceived and very real threat of violence: all these may cause women to keep their HIV status a secret… The fear of knowing and eventually disclosing their HIV status deters women from seeking PMTCT services and results in poor adherence to PMTCT interventions, in particular safer infant-feeding decisions, decisions on taking and adhering to ARV medication, condom use and family planning and preference not to deliver at healthcare facilities’(pg.22–23).*

The National Communication Strategy for the Elimination of Mother to Child Transmission of HIV [eMTCT] (2014–2017) has a section on ‘the role of gender in HIV/AIDS’. The section highlights gender dimensions of women’s vulnerability to HIV/AIDS:

*‘The subordination of African women creates vulnerability to HIV infection through economic dependency, lack of assets, and lack of protection against abuse. …Also social construction of masculinity and femininity renders women powerless to demand for their rights, including not questioning infidelity of their husbands’(pg*.7).

Despite recognition of the gender dimensions of women’s vulnerability to HIV/AIDS, the interventions proposed do not focus on addressing the identified gender bottlenecks. Instead, the communication strategy lists what the key message content for a strategy on male involvement in relation to access and utilization of reproductive and child health (RCH)/PMTCT services should be, without stating how these are to be addressed in ways that address gender norms and affirm gender equality. Message content about men’s role in reproduction and RCH services includes: Knowledge on PMTCT/elimination of mother to child transmission of HIV (eMTCT); Importance of couple HIV testing and counseling (HTC) and Sexually Transmitted Infection (STI) screening in HIV prevention; disclosure and partner care and support; maternal and infant nutrition; ARV adherence; and Family planning methods. (MoHSW 2014:37). The above content focuses on what male attendees [those who comply] should be told, and misses out men who defy calls to take part in PMTCT. The non-attendees are held back by deeply rooted masculine norms which should be targeted in order to increase men’s participation in PMTCT in ways that affirm women’s reproductive rights.

##### Level 4: Gender specific policy/strategy documents

To be rated at GRAS level four the documents had to go beyond indicating gender awareness to state measures for addressing specific concerns of women and/or men and address gender inequity. This was the case only for the National Training Refresher Package: Services for Comprehensive Care and Prevention of Mother-to-Child Transmission, Participant Manual, 2013 (MoHSW, 2013b). Within Unit 1.2, under a section titled: ‘challenges of couples HIV testing and counseling’, a number of gender-related challenges are highlighted, including: low participation of men in RCH services; fear that HIV testing and counseling may result in domestic violence and/or separation/divorce; and the fact that 3–15% of women who disclose their HIV+ status to their male partners experience negative reactions including blame, abandonment, anger, and violence (pg.30).

Besides showing gender awareness (a GRAS level 3 indicator), the document signifies a higher level of gender responsiveness through, for example, stating in the same section:

‘*during counselling health care workers (HCWs) can support the importance of the partner’s presence and stress the man’s responsibility for protecting the health of his wife or partner and their family*. …*Testing both partners together may reduce the chances that the woman will be“blamed”for bringing HIV infection into the family’(*MoHSW 2013, *pg.29).*

This intervention by a health worker helps to overcome a perception that pregnancy, childbirth, and post-delivery child care are concerns for women alone. It provides an opportunity to moderate relationships between partners in decision-making power with regard to: condom use for prevention HIV between partners, use of antiretroviral drugs (ARVs), delivery at the health facility, and utilization of household resources for safe infant feeding. Overall, couple counselling enhances joint family efforts to prevent transmission of HIV to each other and to the child during pregnancy, childbirth, and after delivery. While actions recognizing gendered vulnerabilities are suggested, the document falls short in that it does not emphasize the need to transform gender relations so that women’s reproductive rights are affirmed.

### Gender responsiveness of leadership processes and practices in PMTCT service delivery

With respect to leadership processes and practices, the study examined gender responsiveness in provision of PMTCT services in government facilities. In essence, the study sought to identify gender-related barriers to, and enablers of, provision of PMTCT services at the health facility level. The findings showed that while respondents were generally gender sensitive (GRAS level 3), they tended to have a narrow understanding of the meaning of gender mainstreaming, often equating it with couple antenatal clinic attendance. One interviewee, for example, stated that gender mainstreaming refers to: ‘*attending to both female and male partners so that they both have higher level of correct understanding of how HIV is transmitted from mother to child’(Head of Unit, Health Facility A).* Despite being aware of the influence that gender has on reproductive and child health, respondents did not know of appropriate actions in response to challenges posed by gender norms and decision-making power disparities between women and their male partners.

In the next section, we organize findings around the four elements of a comprehensive approach to PMTCT, as indicated in the National Guidelines for Comprehensive Care Services for Prevention of Mother to Child Transmission of HIV and Keeping Mothers Alive, 2013. They are discussed in turn below.

#### Limited efforts for enhancing primary prevention of HIV among women of childbearing age and their partners

Primary prevention of HIV among women is the most effective means for protecting children against HIV. That is, the risk of MTCT is non-existent when women remain HIV negative. In the current study, almost all respondents claimed their institutions have a system for encouraging men to attend voluntary counselling and testing (VCT) together with their partners. It was further asserted that the set-up of VCT room(s) ensures privacy and confidentiality for the couples. In addition, clients who attend as couples are given priority by allowing them not to stay in a queue. These organizational arrangements provide an opportunity for partners to strategize how to protect both of them—or one of them in case of discordance—from being infected with HIV. For instance, through guidance of the counsellors, partners may resolve to practice safe sex. One of the leaders succinctly described readiness of the health facilities in handling couple VCT:

*Our health facility is well positioned to handle couple VCT. Firstly, we have a local arrangement of favouring couples; secondly, our counsellors are well trained on how to handle couples. Thirdly, the counselling room ensures the highly needed privacy for deciding on safer sex options.* (Head of Unit, Health Facility C).

However, the findings indicate that effectiveness of the joint decision to practice safe sex depends on how well gender concerns are addressed.

Although men and women attending VCT together create the need for staff to introduce more gender sensitive attitudes and approaches to HIV, respondents stated that staff training on how to handle non-clinical gender related challenges—including among counsellors—is virtually non-existent. According to respondents, such challenges include: women’s weak decision-making power on when and how to have sex in a safe way; the weak position of women in negotiating condom use; and, masculine norms that promote infidelity, non-use of condoms, multiple sexual partners, and sexual violence. Examples of the limitations of the training are below.

Our training is limited to those issues that the health system may influence, like facilitation of disclosure of HIV positive status when both partners come to the health facility. However, those women whose male partners cannot or refuse to attend with them, we as the health care providers do not do much. There are a lot of social barriers involved. In the training sessions, health workers are urged to just encourage such women to try hard and disclose but fall short of helping them how to overcome the barriers (Head of Unit, Health Facility C).

Another leader of a health facility added that:

Given limited time that a counsellor has, she/he is not trained to handle distant issues, such as weaker position of the woman in negotiation of safer sex or men’s attitudes that promote engagement in unsafe sex practices. All they do is to advise on safe sex. (Head of Unit, Health Facility B) .

The leaders admitted that the problem is compounded by the absence of guidelines on how to address the above gender-related challenges. For instance, the existing guidelines are silent on how to handle masculine norms that increase vulnerability to HIV, which negatively influence primary prevention of HIV among women.

#### Limited gender consideration in facilitating prevention of unintended pregnancies among women living with HIV

Use of contraceptives is part of a comprehensive approach toward stopping transmission of HIV from the mother to the child. That is, women living with HIV and their male partners should plan when to have a child so that they have time to reflect on the implication of child birth and appropriate measures for PMTCT. Respondents stated that health facilities attempt to encourage women to attend appointments with their partners through health education at family planning (FP) clinics. However, according to some respondents, limited space at FP clinics prevents men from attending. As a result, the set-up is unconducive for couples:

*The area where clients sit waiting for services, has no enough space for couples tosit (Head of Unit, Health Facility B)*.

Furthermore, respondents reported that there are no training programmes for enabling staff to address power imbalances that inhibit women’s decision-making about their fertility. In addition, according to respondents, there are no information packages/guidelines that address the responsibilities of men in family planning.

#### Limited efforts for enhancing male involvement in prevention of vertical transmission of HIV from mothers to their infants

The prevention of vertical transmission of HIV from mothers to their infants is a central element to PMTCT services. It implies provision of a range of services aiming at stopping transmission of HIV from the mother to the child. These include: counseling and testing for pregnant and breastfeeding women and their partners; anti-retroviral therapy (ART) and prophylaxis, safer delivery practices, and counseling on infant feeding and care of the HIV-exposed infant.

Respondents reported that hospitals/clinics use several ways to facilitate couple antenatal clinic (ANC) attendance (including HIV testing). These included: asking first visit pregnant women to invite their partners and issuance of letters of invitation. Similarly, after delivery, mothers are encouraged to attend the postnatal clinic (PNC) together with their spouses, during which a follow up is made on infant feeding. However, according to respondents, male involvement in ANC and PNC is low:

I tell you very few fathers come to the post-natal clinic, fewer than those who attend ANC. (Head of Unit, Health Facility A).

In addition, respondents reported that there is no staff training on how to handle important gender dimensions which affect the vertical transmission of HIV from mothers to their infants, such as inequality in ownership and control of resources and decision-making power. It was also reported that hospitals/PMTCT units did not have communication materials for encouraging men to consistently attend ANC and PNC. Neither do they have health information package/guidelines that address responsibilities of men in prevention of vertical transmission of HIV:

*There is no formal training on how to handle gender related challenges. Nevertheless, staff do use their wisdom and experience to advise clients on the importance of equitable ownership of resources in relation to PMTCT (Head of Unit*, *Health Facility C).*

#### Lack of extended support services for addressing gender-related factors that impinge on treatment, care and support to women living with HIV and their partners, infants, and families

Provision of treatment, care, and support is important for both reducing the risk of transmission of HIV from the mother to the child and for keeping parents alive and strong so they may take care of the child. According to respondents, in addition to providing anti-retroviral drugs (ARVs), health staff educates clients on the importance of disclosing HIV status and of adherence to treatment. Those who cannot adhere because of economic challenges that result in food insecurity are referred to an organization which provides food support.

According to respondents, there are no extended support services for addressing stigma, neglect, gender-based violence (GBV), and abandonment. Similarly, there is no system for following up HIV exposed infants to facilitate sustainability of the chosen safe feeding option. This was compounded by the fact that staff training is to a large extent limited to the clinical perspective, omitting social determinants of adherence. One of the interviewees explained that:

Training of staff on treatment adherence focuses on ARVs adherence, dosage, types of drugs, side effects, and patient follow ups. We do not have care programs at family level (Medical officer in-charge, Health Facility B).

Since staffs are mainly prepared to handle clinical aspects of care and treatment, the support component suffers—particularly on broader issues such as GBV which in turn negatively impacts on PMTCT.

## Discussion

This study has found out that level of gender responsiveness of PMTCT policy/strategy documents varies, whereby some are at GRAS level 3 (gender sensitive), and others are at GRAS level 4 (gender specific). With respect to leadership processes and practices, the study has shown that while respondents were generally gender sensitive (GRAS level 3), they tended to have a narrow understanding of the meaning of gender mainstreaming, often equating it with couple antenatal clinic attendance. Furthermore, although leaders of PMTCT units seemed to be aware of the influence that gender has on reproductive and child health, they did not know of appropriate actions in response to challenges posed by gender norms and decision-making power disparities between women and their male partners. Discussion of these findings follows.

### Limited gender responsiveness of Tanzania’s PMTCT policy/strategy documents

Most policy/strategy documents are gender sensitive, that is, they indicate gender awareness without stating remedial measures. This might be explained by the fact that in the design stage of a policy or strategy, gender is handled as an ‘add on’ rather than being made an integral part of the entire policy/strategy (WHO 2011; Quay and Crawford 2012).

The reasons gender is often treated as an add-on are not very clear. Nevertheless, field experience in development or review of several reproductive health policy/strategy documents shows that the need to integrate gender often does not emanate from within, but rather it is a response to external concerns (Quay and Crawford 2012), such as donor requirements. This is particularly the reality in low income countries which are economically dependent on external bilateral and multilateral donor agencies. Consequently, gender is merely ‘added’ or ‘included’ in order to fulfil certain requirements, instead of making it [gender] an integral part of policy/strategy in order to realize increased effectiveness and coverage. In addition, limited capacity in gender mainstreaming among policy makers may also shed light on why gender is often handled as an add-on.

Thus, although gender features as one of the guiding principles in the introductory chapter(s) of key PMTCT policy documents in Tanzania, gender mainstreaming is low as document goals, objectives, and strategies do not incorporate gender considerations. If such considerations are not made, implementation and evaluation activities will also have very limited consideration of gender.

In the current review of policy documents, consideration of gender was to a large extent confined on promotion of couple voluntary counselling and testing (CVCT)—arguing that it (CVCT) may reduce the chances of the woman being ‘blamed’ for bringing HIV infection into the marriage. However, a number studies (Larsson et al. 2010; Tabana et al. 2013) have suggested that testing partners together without skilfully integrating masculine norms of male dominance in counselling may compromise the intended positive outcome of minimizing the blame on women in case both test HIV positive. For instance, failure to address such masculine norms may explain conflicts and even violence that follow disclosure of HIV positive status during pregnancy and/or postpartum period (Stöckl et al. 2010).

Limited gender integration was also noted in the Ugandan policy/strategy documents (NCG Uganda 2012). This deficiency seems to be a global phenomenon as an international review conducted by Ravindran and Kelkar-Khambete (2007: 4) concluded that: ‘National health programmes that have gender integrally woven into their objectives and activities are rare’. This might be explained by the fact that often the intention is not to take gender concerns on board in implementation of the policy or strategy, but rather indicating acknowledgement of the role of gender in problem causation (Ravindran and Kelkar-Khambete 2007). This is evidenced by the absence of content related to gender in the objectives, strategies, and/or evaluation plan. Consequently, little or no efforts are made to analyze and transform gender-based norms and power inequalities that impinge on PMTCT (Quay and Crawford 2012).

### Predominance of clinical focus limits breadth of gender mainstreaming attempts

The findings show that solutions for addressing gender concerns have been directed at the health facility, while community level efforts are virtually lacking—such as the promotion of couple counseling and testing for HIV. While important, focusing on couple counseling and HIV testing alone is inadequate. Moreover, the documents lack equally important measures that might be implemented outside the health facility targeting barriers such as: women’s economic dependence, lack of decision-making power on sexual and reproductive health, masculine norms that encourage male dominance, and gender based violence. These findings suggest that community based initiatives for PMTCT in Tanzania are lacking. Nevertheless, community based interventions targeting some of these gender-based barriers to PMTCT uptake have been reported elsewhere (Ghanotakis et al. 2012; Clouse et al. 2014). For instance, Engender Health has been implementing the Men as Partners (MAP) program in South Africa and Kenya with a focus on promoting discussion of gender norms and power dynamics that have a bearing on gender based violence (Bott et al. 2005).

The limited coverage of the solutions to gender-related barriers might explain why there is a lack of access to and use of PMTCT services in Tanzania. For instance, maternal ARV coverage is 59%, and only 21% of male partners of pregnant women in Tanzania are tested for HIV (MoHSW 2011). This limited focus has been reported in a review of similar documents in Uganda (NCG Uganda 2012), in which it was observed that while most policy documents explained the importance of gender concerns, they fell short of articulating the procedures and strategies that would ensure that remedial action is taken to address and/or transform inequality generated by unequal gender norms, roles, and relations (NCG Uganda 2012). Therefore, Tanzania’s PMTCT guidelines and other strategy documents should be reviewed to address gender concerns much more comprehensively beyond the clinical domain. Additionally, there is a need to establish or strengthen the community component of PMTCT intervention and learn from best practices elsewhere.

### Limited gender responsiveness of leadership processes and practices

Assessment of leadership processes and practices revealed little attention to the integration of gender in the delivery PMTCT services. It was found that strengthening the gender capacities of health service providers is generally absent. Similar findings have been reported in Uganda, where it was reported that indifference at the leadership level resulted in a lack of actions for addressing gender-related challenges against PMTCT and staff were not held accountable for their inactions (NCG Uganda 2012). Likewise, in Kenya, assessment indicated that there were no specific strategies for addressing stigma and discrimination among HIV infected postpartum women (NACC 2002).

Limited integration of gender at service delivery points is a reflection of gaps in the guidelines. That is, health staff with limited grounding on gender mainstreaming and who use guidelines with low level of gender responsiveness may fail to recognize gendered PMTCT challenges and consequently may not take appropriate actions. In sum, limited incorporation of gender into PMTCT policy and practice in Tanzania implies that the powerful impact that gender has on effectiveness and coverage of the PMTCT programs has not been well appreciated. Consequently, the goal of getting to zero HIV infection among infants will remain out of reach. This calls for programme review and capacity strengthening at service delivery points. There is need for health managers and staff to be equipped with the knowledge and skills for addressing gender-based health inequities—including those related to PMTCT.

### The role of health workers in gender mainstreaming of PMTCT: a missed opportunity

The findings of this study suggest that limited gender mainstreaming of policy documents and leadership practices confine health workers on the clinical domain of PMTCT. This constitutes a missed opportunity toward comprehensive handling PMTCT in the health care facility. Much as social aspects of PMTCT cannot be exhaustively addressed within the health care setting, the health worker is better placed to enable his/her clients to understand the linkage between gender and PMTCT through health education andindividual/couple counselling sessions (WHO 2009). Through these sessions, women and their male partners can critically reflect on how gender norms shape the fate of the new-born in the context of HIV transmission. Specifically, the couples may reflect on the influence of masculine norms and decision-making power disparity on: clinic attendance, adherence to anti-retroviral drugs, and adherence to safe infant feeding options. Additionally, health workers are well placed to conduct similar sessions in the community setting through outreach programmes; and they can appropriately refer individuals/couples to community based gender transformative initiatives. Suitability of the health workers in explaining social aspects of transmission of mother to child trans mission of HIV is due to both their technical capacity and the trust that people have in them. Nevertheless, health workers themselves will need appropriate gender training to be able to carry out these roles. Key gender training areas may include: understanding the influence of masculine norms on clinic attendance and adherence to safe infant feeding option; recognizing GBV and appropriate interventions; and utilization of gender sensitive protocols for counseling. The WHO’s publication ‘Integrating gender into HIV/AIDS programmes in the health sector: Tool to improve responsiveness to women’s needs’ is a very good resource (WHO 2009).

### Study limitations

This paper is based on a study conducted in one city in Tanzania, using a qualitative approach. This limits generalization. However, since policy documents are national instruments—the findings have national implications for improvement. Additionally, since the study involved conducting interviews, another limitation was recall bias. The respondents might have experienced difficulty in remembering some facts regarding leadership processes and practices. This was mitigated by asking focused questions and the interviewer checked if the questions were well understood.

## Conclusion

The study has revealed limited integration of gender concerns (less or lack of attention on the disadvantageous position of women in terms of inequality in ownership of resources, power imbalance in decision-making, asymmetrical division of roles, and masculine norms that distance men from maternal and child care) in PMTCT policy documents. Similarly, health facility leaders pay little attention to the integration of gender in the delivery of PMTCT services. Deficiency in leadership practices reflects gaps in the guidelines. This may partly be attributed to the predominant focus of PMTCT programme on clinical services. The gaps in both guidelines and leadership at service delivery level makes it difficult to effectively deliver PMTCT services and to monitor programme implementation from a gender perspective. Revision of guidelines and strengthening of gender capacities of leadership at service delivery level are greatly needed. Ethical approval was obtained from the authors’ Institute.
